# Actin from within – how nuclear myosins and actin regulate nuclear architecture and mechanics

**DOI:** 10.1242/jcs.263550

**Published:** 2025-02-10

**Authors:** Marta Gawor, Lilya Lehka, Danielle Lambert, Christopher P. Toseland

**Affiliations:** ^1^ Laboratory of Molecular Basis of Cell Motility, Nencki Institute of Experimental Biology, Polish Academy of Sciences, 3 Pasteur St., 02-093 Warsaw, Poland; ^2^ Division of Clinical Medicine, School of Medicine and Population Health, University of Sheffield, Sheffield S10 2RX, UK

**Keywords:** Myosin, Actin, Nucleus, Mechanobiology

## Abstract

Over the past two decades, significant progress has been made in understanding mechanotransduction to the nucleus. Nevertheless, most research has focused on outside-in signalling orchestrated by external mechanical stimuli. Emerging evidence highlights the importance of intrinsic nuclear mechanisms in the mechanoresponse. The discovery of actin and associated motor proteins, such as myosins, in the nucleus, along with advances in chromatin organisation research, has raised new questions about the contribution of intranuclear architecture and mechanics. Nuclear actin and myosins are present in various compartments of the nucleus, particularly at sites of DNA processing and modification. These proteins can function as hubs and scaffolds, cross-linking distant chromatin regions and thereby impacting local and global nuclear membrane shape. Importantly, nuclear myosins are force-sensitive and nuclear actin cooperates with mechanosensors, suggesting a multi-level contribution to nuclear mechanics. The crosstalk between nuclear myosins and actin has significant implications for cell mechanical plasticity and the prevention of pathological conditions. Here, we review the recent impactful findings that highlight the roles of nuclear actin and myosins in nuclear organisation. Additionally, we discuss potential links between these proteins and emphasize the importance of using new methodologies to unravel nuclear-derived regulatory mechanisms distinct from the cytoskeleton.

## Teaser

**Summary:** A review of how nuclear actin and myosins regulate nuclear shape and mechanics through intranuclear organisation, chromatin remodelling and nuclear mechanopathways, which has significant implications in cell adaptation and pathology.

## Introduction

Cell adaptation to mechanical stimuli requires coordinated modifications of membranes, the cytoskeleton, organelles and intracellular compartments. The speed and synergy of the mechano-response of the cell are crucial for maintaining homeostasis and plasticity. Impairment of these processes leads to pathology and disease (Gaetani et al., 2020; Verbruggen, 2018). As the most prominent and stiffest organelle in the cell, the nucleus serves as a major mechanosensing platform that impacts cellular mechanics (Aureille et al., 2019; Fischer et al., 2020; Kirby and Lammerding, 2018). Consequently, nuclear shape is often used as a hallmark of the overall biophysical state of the cell and as a marker for diseases such as Hutchinson–Gilford syndrome (progeria) and cervical, prostate and breast cancer, as well as dilated cardiomyopathy (Lu et al., 2018a; Malshy et al., 2022).

The nucleus is a highly organised organelle that stores genetic information and houses the initial stages of gene expression. Distinct nuclear compartments include the nucleolus, where ribosomes are formed and ribosomal RNA is transcribed, chromosomes, which package DNA to ensure its accurate replication and distribution, chromosome territories, which are specific subnuclear regions occupied by chromosomes, and nuclear speckles and bodies, which are subnuclear chromatin domains enriched with RNA processing machinery (Cremer et al., 1993; Razin et al., 2014; Strouboulis and Wolffe, 1996). The nucleus is surrounded by a mesh of actin, intermediate filaments and microtubules, which collectively maintain nuclear shape and integrity and ensure proper nuclear localisation within the cytoplasm. The morphology of the nucleus is regulated on multiple levels by factors including forces transmitted through the cytoskeleton, the thickness and tension of the nuclear lamina (a fibrillar network which surrounds the nucleus), chromatin compaction and the local conformation and mobility of chromatin (Dahl et al., 2008; Lele et al., 2018). Aberrations in nuclear shape are often accompanied by changes in chromatin organisation, which in turn affect transcription, DNA repair and mRNA transport, among other processes (Pfeifer et al., 2018; Pfleghaar et al., 2015). Although nuclear mechanobiology research traditionally focuses on outside-in signalling and regulation of the nucleus, a growing body of evidence suggests that inside-out signalling from the nucleus, along with internal mechanical regulation, plays a significant role in cellular homeostasis. For instance, genome reorganisation and changes in nuclear integrity can reshape the cytoskeleton. Exploring the functionality of nuclear actin and myosin within these processes therefore provides an interesting perspective. Understanding the interplay between nuclear architecture, integrity and genome organisation, and their impact on cellular mechanics is crucial for understanding and tackling pathologies, including cancer, viral infection and congenital disorders.

In this Review, we discuss recent advances in nuclear actin and myosin research and their potential impact on nuclear mechanobiology, focusing on changes in nuclear architecture and genome organisation. We summarise the most impactful findings, examine the current understanding of the nuclear actin–myosin relationship and outline future directions for this field.

## Mechanotransduction to the nucleus

The biophysical features of the extracellular matrix (ECM) profoundly impact cell physiology and adaptation (Di et al., 2023). The contact between ECM and the actin cytoskeleton is mediated by various receptors, including integrins (Hynes, 2002). Talin and vinculin, which interact with both integrins and filamentous actin (F-actin), transmit ECM-derived and intracellular forces generated by myosin II and retrograde actin flow to the ECM (Case et al., 2015; Kanchanawong et al., 2010). Mechanoactivation triggers a cascade of multilevel responses, enabling cells to adapt to changing conditions, reflected in their morphology and behaviour.

**Fig. 1.**
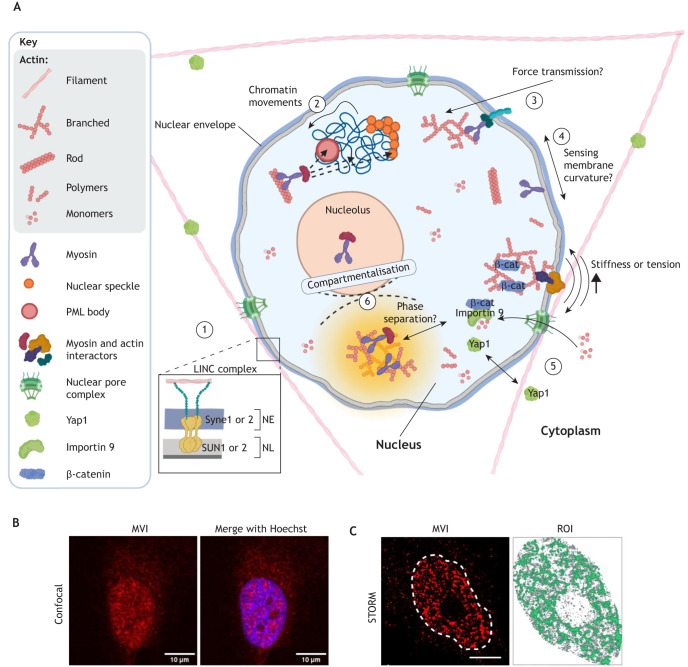
**Nuclear myosin and actin in the nucleus.** (A) Potential mechanosensitive pathways engaging nuclear actin and myosins. (1) The LINC complex connects the nuclear envelope (NE) and nuclear lamina (NL), facilitating force transmission. The scheme depicts an example of the possible LINC complex consisting of SUN1 or SUN2 and Syne1 or Syne2. (2) Nuclear actin and myosins within chromatin domains (dashed arrows) reorganise chromatin. (3) Force-sensitive nuclear motors have a potential role in transmitting forces within the nucleus. (4) MVI senses membrane curvature; however, it remains unclear whether it performs this function within the nucleus. (5) Dynamic strain stimulates importin-mediated nuclear transport of actin and β-catenin but does not impact Yap1 nuclear translocation. (6) Nuclear myosin and actin contribute to compartmentalisation, including phase separation (dashed lines). (B) Visualisation of MVI. An example confocal microscopy image of MVI immunostaining in HeLa cells, where nuclear MVI is predominantly located outside the nucleolus. A merge with Hoechst staining is shown on the right. Scale bars: 10 μm. (C) Visualisation of MVI. A stochastic optical reconstruction microscopy (STORM) render of nuclear MVI in HeLa cell (left) and a region of interest (ROI) (dashed lines). Scale bar: 5 μm. A cluster map of the selected ROI (right) reveals that MVI molecules are distributed in clusters (green), with over 70% of MVI molecules typically present within clusters (Hari-Gupta et al., 2022; Pageon et al., 2016). Images in B and C by D.L. Created in BioRender by Gawor, M., 2025. https://BioRender.com/u29u030. This figure was sublicensed under CC-BY 4.0 terms.

The main connector and force transmitter between the nucleus and the cytoskeleton is the linker of nucleoskeleton and cytoskeleton (LINC) complex, comprising SUN-domain (SUN1 and SUN2) and KASH-domain (Syne1–Syne4, KASH5 and KASH6) proteins (Fig. 1A). The LINC complex connects the nucleus to the perinuclear actin cap, an actomyosin structure that covers the top of this organelle and generates contractile forces that reshape the nucleus. Forces transmitted through the LINC complex at the nuclear envelope stretch the nuclear pore complex (NPC), impacting the nuclear shuttling of mechanosensitive proteins, such as the Yes-associated protein 1 (Yap1) (Elosegui-Artola et al., 2017; Scott et al., 2021; Strambio-De-Castillia et al., 2010). Mechanotransduction on the nucleus can also occur via alternative mechanisms, including the contraction of central actin stress fibres, movement of cell boundaries, geometric constraints imposed by excess nuclear lamina, and nuclear compression occurring during cell reshaping and movement (Dickinson et al., 2022; Dickinson and Lele, 2023; Jahed and Mofrad, 2019). Furthermore, nuclear shape fluctuates subtly on a timescale of seconds, with these undulations decreasing as the cell cycle progresses (Chu et al., 2017; Hennen et al., 2020; Introini et al., 2023). Although the significance of these subtle changes remains unknown, they highlight the presence of mechanisms that constantly orchestrate the cellular response to micro-stimuli and are potentially distinct from canonical pathways controlling more pronounced shifts in cell morphology.

## Actin and myosin within the nucleus

The rapidly developing field of nuclear actin and myosin research provides mounting evidence of the links between chromatin structure and the activity of these proteins, with potential implications for cellular mechanics. Actin is present in the nucleus in various forms, including globular (monomeric) actin (G-actin), short filaments (rods), condensates or polymers (Fig. 1A) and has been shown to participate in chromatin remodelling, chromosome movement, transcription and RNA processing and turnover (Chuang et al., 2006; Hofmann et al., 2004; Philimonenko et al., 2004; Sanger, 1975; Szerlong et al., 2003; Ulferts et al., 2024; Viita et al., 2019). The discovery of nuclear actin was accompanied by the identification of several types of nuclear myosins, including nuclear myosin I (NMI; heavy chain MYO1C), non-muscle myosin II (NMII; heavy chains MYH9 and MYH10), and myosins V (MV; heavy chains MYO5A–MYO5C), VI (MVI; heavy chain MYO6), X (MX; heavy chain MYO10), XVI (MXVI; heavy chain MYO16) and XVIII (MXVIII; heavy chains MYO18A and MYO18B) (Barisic et al., 2019; Lane, 1969; Nowak et al., 1997; Ohnishi et al., 1963; Pestic-Dragovich et al., 2000; Shahid-Fuente and Toseland, 2023; Simon and Wilson, 2011; Tolstonog et al., 2002; Traub and Shoeman, 1994; Venit et al., 2020a). Similar to what is seen for actin, nuclear myosins are engaged in intranuclear transport, transcription, splicing, chromosome movement and DNA damage repair (Cook et al., 2020; de Lanerolle, 2012; Hurst et al., 2019). Myosins are distributed throughout the nucleus and in nuclear bodies, speckles and euchromatin, where they regulate chromatin organisation (Fig. 1A) (Majewski et al., 2018; Venit et al., 2020a). Evidence shows that myosins are present in dense protein clusters like other active nuclear proteins, such as RNA polymerase II (RNAP II), suggesting that they have coordinated or synchronised activity (Hari-Gupta et al., 2022). The multifunctionality of myosins arises from multiple regulatory mechanisms influenced by processes, such as alternative splicing, phosphorylation, auto-inhibitory conformations, interactions with binding partners, applied mechanical load, phosphoinositide signalling or divalent cation concentration (Buss et al., 2001; Fili et al., 2020; Fili and Toseland, 2019; Finan et al., 2011; Komaba et al., 2010; Laakso et al., 2008; Lu et al., 2011; Niu et al., 2024; Rosenfeld et al., 2005). This multilevel regulation supports the idea that myosins contribute to both immediate and long-term nuclear responses, potentially via mechanisms distinct from those in the cytoplasm.

The extent to which nuclear actin and myosins cooperate and whether their nuclear structures are functionally comparable to those in the cytoplasm remain open questions. The unique intranuclear environment – characterised by limited space, densely packed chromatin, proximally located differentially condensed regions and the need for finely tuned local regulation of chromatin dynamics – likely requires a structurally flexible and specialised network. Importantly, actin and myosins can execute their nuclear function independently or in complexes. For instance, NMI is necessary for RNAP II to generate the first phosphodiester bond during transcription initiation but is not required for formation of the RNAP II pre-initiation complex, whereas β-actin is indispensable for the latter (Hofmann et al., 2004, 2006). Conversely, actin and NMI cooperate in ribosomal DNA transcription and chromosome repositioning in response to changing cellular behaviour (Chuang et al., 2006; Dundr et al., 2007; Mehta et al., 2010; Ye et al., 2008). It remains unclear whether myosins can regulate actin organisation in the nucleus as observed in cytoplasmic contexts, such as in spermatids or around mitochondria (Isaji et al., 2011; Kruppa et al., 2018).

Despite increasing insights into the nuclear functions of actin and myosins, little is known about their relationship and role in nuclear mechanics. One major challenge in studying these proteins is their constant nucleocytoplasmic shuttling (Jain et al., 2013; Skarp et al., 2013; Virtanen and Vartiainen, 2017). Conventional molecular tools target total cellular pools of actin and myosins, making it difficult to dissect nucleus-specific functions. This is particularly relevant to treatments using drugs that target the Arp2/3 complex, such as CK666, which impact cytoplasmic actin in addition to the nuclear pool. However, the use of nuclear-specific actin or myosin variants and targeted manipulation of nuclear import or export pathways has enabled more precise studies of their nuclear roles (Hari-Gupta et al., 2022). Nevertheless, the potential for myosins to have multiple nuclear functions complicates efforts to target nuclear myosins in specific processes. Recent developments in imaging technologies, such as atomic force microscopy (AFM), stochastic optical reconstruction microscopy (STORM) and high-content imaging (HCI), as well as studies on isolated nuclei, promise to provide deeper insights into actin and myosin organisation and function in the nucleus (Bond et al., 2022; Dos Santos et al., 2022a,b; Fili et al., 2017; Gunn et al., 2024; Hobson et al., 2020; Lherbette et al., 2017).

Although the roles of actin and myosins in the nucleus have been discussed elsewhere, mainly in the context of transcription and chromatin modifications, their impacts on nuclear biophysical properties are often overlooked (Cook et al., 2020; Greenberg et al., 2016; Kyheröinen and Vartiainen, 2020; Shahid-Fuente and Toseland, 2023). We will next overview the current knowledge on these impacts.

## Actin and myosin as architects of the nucleus

The dynamic connection between cytoplasmic and nuclear pools of actin and myosin raises the crucial question of to what extent their intranuclear forms contribute to force detection and transmission (Fig. 1A). Although nuclear actin has been implicated in reshaping the nucleus after mitosis, its role in nuclear mechanosensing remains elusive (Baarlink et al., 2017; Krippner et al., 2020; Skory et al., 2023).

Interestingly, nuclear NMI and MVI are force-sensitive (Altman et al., 2004; Laakso et al., 2008). For example, class I myosins sense tension in epithelial cells of the intestine and in hair cells in the inner ear (Batters et al., 2004; Holt et al., 2002; Nambiar et al., 2009; Stauffer et al., 2005). Moreover, the affinity of MVI for ADP and ATP and its binding to actin are regulated by mechanical load (Altman et al., 2004). This mechanism enables MVI to switch between roles as a cargo transporter and a protein anchor, supporting nuclear architecture. This myosin-anchoring function is thought to operate in concert with actin (Hari-Gupta et al., 2022). Interestingly, recent findings show that MVI regulates F-actin polymerisation and bundling and crosslinks it into branched contractile networks (Hook et al., 2024). Those authors suggest that MVI-enriched nodes in these networks provide tension and support, protecting the actin structure from deformation. However, further research is needed to determine whether MVI similarly controls nuclear actin assembly.

Another study has revealed that overexpression of F-actin in nuclei from *Xenopus* egg extracts and in HeLa cells impairs nuclear morphology, and this effect is mitigated by the expression of lamin A, a core protein of nuclear lamina (Mishra and Levy, 2022). This finding suggests that lamin counteracts forces generated by nuclear actin to regulate nuclear biophysical properties. Interestingly, the effect of nuclear F-actin is mediated by formins, which are F-actin nucleators, rather than myosins or the actin cytoskeleton regulator Arp2/3, indicating that myosin and actin can function independently to regulate nuclear architecture, as they do in the cytoplasm.

Additionally, MVI can independently regulate membrane sculpting. In a model using reconstituted lipid bilayers and reshaping nanoparticles, MVI binds membranes in a curvature-dependent manner, remodelling them into dynamic geometric patterns controlled by tension and stiffness (Rogez et al., 2019). According to the theoretical model describing this phenomenon, local membrane curvature and MVI binding alone can impact membrane geometry independently of MVI motor activity or actin. The authors propose a mechanism where the binding of MVI to lipids creates dynamic feedback and instability triggering membrane reorganisation. This raises the question of whether MVI or other nuclear myosins interact similarly with nuclear membranes (Fig. 1A). NMI, for example, regulates plasma membrane tension in fibroblasts, although the sub-membranous actin network also plays a role (Venit et al., 2016). Further studies employing various cell models and biophysical techniques are required to explore the involvement of nuclear actin in mechanosensing and to clarify whether nuclear myosins independently sense and regulate cellular and nuclear membrane geometry. Nevertheless, a mechanism relying solely on the detection of membrane curvature is an attractive explanation for rapid local changes in nuclear shape that occur within seconds and could potentially be regulated by myosins.

The nuclear translocation of the Yap1 mechanosensor is mediated by cytoplasmic actomyosin mechanosensing regulated by the LINC complex, lamin A expression, and nuclear shape and stiffness, ultimately leading to the transcription of mechanoresponsive genes (Das et al., 2016; Driscoll et al., 2015; Panciera et al., 2017; Scott et al., 2021). A recent study highlights an additional crosstalk between nuclear actin and Yap1 signalling in mesenchymal stem cells (MSCs) (Fig. 1A) (Sen et al., 2022). That study shows that MSCs respond differently to static and dynamic ECM-derived forces. Dynamic strain, which changes quickly following an applied force, induces nuclear accumulation of β-catenin – a transcriptional co-activator – and actin, increasing cell and nuclear stiffness, but not affecting Yap1 shuttling. Additionally, β-catenin nuclear transport appears to depend on actin (Fig. 1A) (Sen et al., 2022). In contrast, static strain, which changes slowly over time, promotes Yap1 nuclear translocation without affecting β-catenin or nuclear actin levels. Transmembrane actin-associated nuclear (TAN) lines, which are actin stress fibres attached to the nuclear envelope by the LINC complex, might be involved in Yap1 translocation by regulating NPC organisation (Hoffman et al., 2020). However, Yap1 nuclear shuttling can also occur in the absence of F-actin or active myosin, driven solely by nuclear compression (Elosegui-Artola et al., 2017; Furukawa et al., 2017; Hoffman et al., 2020; Koushki et al., 2023). The Yap1 mechano-pathway also interacts with the ataxia telangiectasia mutated (ATM) kinase during mechanical stress responses. ATM, a DNA damage response (DDR) regulator, associates with the actin cytoskeleton and controls the phosphorylation of cytoskeletal and chromatin organisers. ATM depletion results in actin stress fibre accumulation, nuclear flattening and deformation, chromatin condensation and Yap1 nuclear accumulation (Bastianello et al., 2023). Overall, these results suggest that the actin and Yap1 crosstalk is context dependent, highlighting how nuclear shape directly impacts signalling pathways.

How the nuclear actin pool regulates mechanosensors in the nucleus remains an emerging question. One possible pathway involves serum response factor (SRF), a regulator of Yap1 in cancer (Guo et al., 2022; Salem et al., 2023). Nuclear actin controls the localisation and activity of megakaryocytic acute leukemia protein (MAL; also known as MRTFA), an SRF coactivator that binds to SRF target genes, and both nuclear actin and MAL dynamics are altered in the absence of lamin A/C (Ho et al., 2013; Vartiainen et al., 2007). However, whether nuclear actin regulates Yap1 transcription or nuclear retention remains unclear. Interestingly, Yap1 can also regulate actin organisation, as shown in gastric or colorectal cancer cells, where Yap1 impairs both cytoplasmic G-actin and F-actin dynamics (Naktubtim et al., 2022; Qiao et al., 2017). Future research should explore how Yap1 translocation reciprocally affects the nuclear actin pool.

## Actin and myosin as organisers of intranuclear space

Nuclear compartmentalisation profoundly influences the micro- and macro-scale organisation of the nucleus and gene expression (Fig. 1A) (Bhat et al., 2021; Meldi and Brickner, 2011). Importantly, chromatin rearrangements are closely associated with nuclear shape and mechanics and their mis-regulation is linked to cancer and infertility (Damodaran et al., 2019). Nuclear myosins contribute to nuclear organisation by crosslinking, stabilising and anchoring proteins, DNA and RNA, recruiting them to actin and affecting chromatin condensation (Chuan et al., 2011; Cook et al., 2020; Fili et al., 2017; Greenberg et al., 2012; Hari-Gupta et al., 2022). Myosins are found in nucleoli, nuclear speckles, nuclear bodies and sites of chromatin reorganisation, where they play roles in separating sites of RNA processing, transcription and replication (Hari-Gupta et al., 2022; Lindsay and McCaffrey, 2009; Majewski et al., 2018; Nowak et al., 2024; Pranchevicius et al., 2008). Similarly, nuclear actin is involved in forming chromatin functional domains that facilitate DNA repair (Zagelbaum et al., 2023). The implications of actin- and myosin-mediated chromatin reorganisation for nuclear mechanics will be discussed in the next section.

In the past decade, another route of cellular and nuclear compartmentalisation has gained more attention (Aumiller et al., 2014; Banani et al., 2016; Boeynaems et al., 2018; Iborra, 2007). Phase separation, a process leading to selective protein accumulation and the formation of liquid-like droplets, allows these condensates to fuse and expand. This phenomenon is a promising mechanism for regulating cellular and nuclear mechanics. Liquid-like condensates can form rapidly in response to extracellular stimuli, such as changes in protein concentration or solvent conditions (Alberti et al., 2019; Krainer et al., 2021). These changes are potentially connected to cellular and nuclear mechanical responses. For example, phase separation drives heterochromatin formation, which increases nuclear stiffness (Larson et al., 2017; Strom et al., 2017). Given that nuclear flattening caused by the actin cap can increase nuclear envelope tension and impact chromatin architecture, it might also reorganise protein droplets (Davidson and Cadot, 2021). Phase separation is important for compartmentalisation, but the roles of nuclear actin and myosins in this process remain poorly understood (Fig. 1A) (Feric et al., 2016). Emerging studies suggest that liquid-like droplets regulate actin polymerisation, balancing branching and bundling to influence cellular shape and movement (Graham et al., 2024). In *Xenopus* oocytes, disruption of nuclear F-actin causes ribonucleoprotein droplets, including nucleoli and histone locus bodies, to sediment and fuse, resulting in a more viscous nucleus (Feric and Brangwynne, 2013). Understanding how actin organisation impacts phase separation and the role of chromatin in this mechanism requires further investigation. Recent findings highlight the connection between the nuclear actin pool, phase separation and transcription (Knerr et al., 2023), showing that the cytoskeletal organiser dishevelled associated activator of morphogenesis 2 (DAAM2) stimulates nuclear actin polymerisation and the formation of actin-dependent protein droplets enriched with active RNAP II. This supports the hypothesis that nuclear actin assembly promotes the formation of transcriptionally active sites through phase separation. Given the stabilising, cross-linking, recruiting and anchoring roles of nuclear myosins, they are likely involved in pathways controlling nuclear compartmentalisation, including phase separation. For instance, nuclear MVI anchors RNAP II in high-density clusters in an actin-dependent manner and such clustering supports phase separation (Hari-Gupta et al., 2022; Lu et al., 2018b; Palacio and Taatjes, 2022). Representative images of MVI distribution into clusters in the nucleus are shown in Fig. 1B,C. This finding suggests that MVI and actin promote the formation of liquid-like droplets with high molecular density. However, the direct involvement of these proteins in phase separation remains to be elucidated.

## Actin and myosin in chromatin organisation and nuclear mechanics

**Fig. 2.**
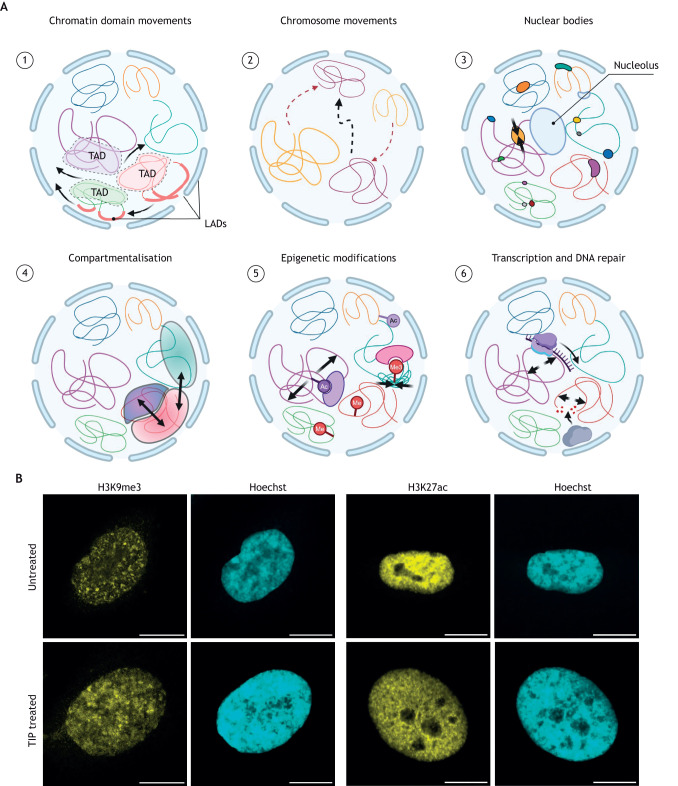
**Chromatin organisation impacts nuclear mechanics and is regulated by nuclear myosin.** (A) Chromatin organisation and nuclear mechanics. (1) Local chromatin movements within lamina-associated domains (LADs) and topologically associated domains (TADs) (dashed areas) are coordinated with nuclear lamina remodelling (arrows). (2) Homologous (black arrow) and non-homologous (red arrows) long-distance chromosome movements cause wide-range reorganisation of the nuclear envelope. (3) Nuclear bodies, such as the nucleolus or nuclear speckles, as well as chromatin clusters (coloured round shapes) serve as sites for local protein recruitment and condensation, changing chromatin compaction (arrows). (4) Chromatin reorganisation into domains and nuclear bodies (coloured areas) enables compartmentalisation of the nucleus (arrows), promoting the separation and precise regulation of specific molecular processes. Colour intensity increases with chromatin compaction. Nuclear lamina stiffness differs between compartments. (5) Epigenetic marks regulate local chromatin compaction by opening or closing chromatin structure (arrows). Ac, acetylation; Me, methylation. (6) Changes in chromatin architecture by epigenetic regulators enable transcription and DNA repair, which further impact nuclear mechanics (arrows). Transcribing RNA polymerase is represented by the violet shape and ligase repairing DNA by the grey shape. (B) Example immunofluorescence staining of histone marks (yellow) and DNA (blue) in HeLa cells under untreated and TIP-treated conditions. Scale bars: 10μm (untreated) and 5 µm (TIP). TIP, an inhibitor of MVI, alters histone marks. Histone H3K27ac, a positive epigenetic mark associated with active gene expression, decreases following TIP treatment. Conversely, H3K9me3, a mark of transcriptional repression, increases under TIP-treated conditions (Hari-Gupta et al., 2022). Images in B by D.L. Created in BioRender by Gawor, M., 2025. https://BioRender.com/u10m947. This figure was sublicensed under CC-BY 4.0 terms.

Within the mammalian nucleus, chromosomes occupy distinct domains. Typically, gene-poor chromosomes are localised near the nuclear envelope and anchored to the lamina in lamina-associated domains (LADs) (Fig. 2A) (Croft et al., 1999). This arrangement forms densely packed chromatin, termed heterochromatin. Conversely, gene-rich chromosomes reside centrally within the nucleus (Ranade et al., 2019) in a more open and relaxed state, known as euchromatin. These regions are dynamic, allowing for long-range chromosome movement, chromatin interactions and DNA diffusion within and between regions (Fig. 2A) (Chung et al., 2023; Hildebrand and Dekker, 2020; Mazzocca et al., 2023). Chromosome structures are further divided into topologically associated domains (TADs) (Fig. 2A) (Buchwalter et al., 2019; Venit et al., 2020a). Changes in chromatin nuclear position and TADs occur in response to perturbations, such as signalling activation, mechanical alterations and DNA damage, facilitating alterations in gene expression or DNA repair mechanisms. These mechanisms of chromatin organisation significantly influence nuclear mechanics. For example, an increase in euchromatin through decondensation softens the nucleus (Fig. 2A) (dos Santos et al., 2021; Dos Santos and Toseland, 2021; Lherbette et al., 2017). Nuclear myosins, such as MVI, regulate chromatin architecture through epigenetic modifications on histones. MVI inhibition decreases chromatin transcriptional activation marks, such as H3K27ac, while increasing transcriptional repression marks, such as H3K9me3 (Fig. 2B) (Hari-Gupta et al., 2022).

**Fig. 3.**
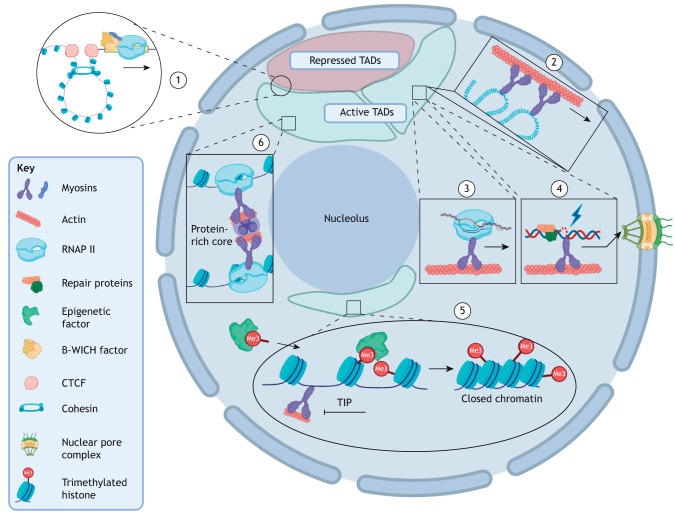
**Role of nuclear actin and myosin in chromatin organisation and its impact on nuclear mechanics.** (1) Regulation of short-range TAD movements by NMI. NMI interacts with the B-WICH complex, which recruits the CTCF DNA-binding protein. CTCF, together with cohesin, regulate loop extrusion and establishes the boundaries and spatial localisation of TADs. (2) Facilitation of long-range chromosome movements. NMI, MVI and actin participate in long-range chromosome movements along actin filaments, enabling large-scale chromosomal reorganisation. (3) Chromatin reorganisation during transcription. Nuclear myosins (NMI and MVI) and actin regulate chromatin reorganisation during transcription. NMI and MVI anchor RNAP II at transcription sites, where the resulting open chromatin indirectly impacts nuclear mechanics. (4) Chromatin reorganisation during DNA repair. Nuclear myosins are implicated in chromatin reorganisation during DNA repair. NMI and MV function as transporters, whereas MVI is likely an anchor, tethering damaged DNA to actin filaments for efficient repair. (5) Recruitment of epigenetic factors. Nuclear myosins and actin recruit epigenetic factors that regulate chromatin compaction. Inhibition of MVI–actin interaction using 2,4,6-triiodophenol (TIP) alters the chromatin epigenetic landscape and chromatin organisation (Hari-Gupta et al., 2022). (6) Nuclear myosins contribute to chromatin stabilisation and crosslinking. During transcriptional gene pairing, MVI anchors RNAP II and crosslinks chromatin around a protein core consisting of transcriptional regulators and coated with actin. This crosslinking reduces chromatin mobility, thereby increasing nuclear stiffness, impacting nuclear mechanics. Created in BioRender by Gawor, M., 2025. https://BioRender.com/c00i452. This figure was sublicensed under CC-BY 4.0 terms.

Altogether, myosins impact pathways involved in DNA damage repair, chromatin structure regulation, chromosome movement and transcription, thereby impacting genome organisation in diverse and specific ways (Fig. 3) (Barisic et al., 2019; Caridi et al., 2018; Cook et al., 2020; Cook and Toseland, 2021; Fili et al., 2017; Hari-Gupta et al., 2022; Hurst et al., 2019; Mehta et al., 2021; Venit et al., 2020a). Therefore, myosins likely contribute directly and indirectly to nuclear mechanics. NMI interacts with chromatin remodellers to facilitate chromosomal movement and chromatin rearrangements associated with transcription and DNA damage repair (Fig. 3) (Venit et al., 2020a). NMI-dependent chromatin movement has been linked to actin, although the exact mechanism remains unclear (Chuang et al., 2006). For instance, chromosome 10 displays altered positioning during senescence in human dermal fibroblasts (HDFs), localising to the nuclear interior rather than the periphery. Stimuli such as serum removal or heat shock can induce chromosome repositioning, but chromosome 10 remains unresponsive during senescence (Mehta et al., 2021). This coincides with a redistribution of NMI, which forms large nucleoplasmic aggregates during senescence, potentially sequestering chromosome 10 and impairing its ability to reposition (Mehta et al., 2021). Chromosomal territory alterations are also observed upon DNA damage, and NMI perturbation halts these reorganisations (Kulashreshtha et al., 2016). Notably, double-stranded breaks cause damaged TADs to cluster, forming new chromatin compartments (Arnould et al., 2023). Such clustering may occur within or between chromosomes. At smaller scales, NMI impacts TAD organisation by interacting with the ISWI chromatin remodeller SNF2H and the Williams syndrome transcription factor (WSTF; also known as BAZ1B), forming the B-WICH complex. This complex recruits CCCTC-binding factor (CTCF), which determines the genomic localisation of a TAD (Fig. 3) (Barisic et al., 2019; Venit et al., 2020a).

Nuclear actin has also been implicated in long distance movements of specific gene loci (Dundr et al., 2007) and in the relocation of heterochromatin double strand brakes during repair (Fig. 3) (Caridi et al., 2018). This relocation involves motion along actin filaments and the recruitment of the DNA repair complex Unc45–Smc5/6 (the Smc5 and Sm6 heterodimer) by NMI and MV.

The role of MVI in genome organisation primarily relates to gene expression and focuses on activity at the gene level. For example, MVI knockdown significantly reduces the homologous pairing of tumor necrosis factor (*TNF*), *TNFAIP8* and *TBET* (also known as *TBX21*) alleles, which is essential for biallelic gene expression during effector T cell activation (Zorca et al., 2015). MVI also anchors RNAP II at transcription initiation sites, supporting the formation of transcription hubs or factories (Fig. 3) (Hari-Gupta et al., 2022). Similar functions have been described for nuclear actin (Wei et al., 2020; Zhu et al., 2004). These transcription sites coordinate gene expression in response to stimuli such as serum, among others (Hari-Gupta et al., 2022; Wei et al., 2020). Although these studies emphasize MVI functions at the gene level, its perturbation also induces widespread alterations in genome organisation, including increased closed chromatin and repressive histone marks (Fig. 3) (Hari-Gupta et al., 2022). However, these changes might result indirectly from RNAP II perturbation at the gene level. Importantly, the nuclear actin pool has also been implicated in regulating chromatin accessibility through epigenetic modifications (Sen et al., 2024).

Chromosome reorganisations within the nucleus will lead to alterations to nuclear mechanics. However, these changes can be classified as indirect, based on myosin impacting chromosome positioning, and it is the change in position that brings about alterations to mechanics. Conversely, a direct impact on cell mechanics can be determined from ability of MVI to stabilise gene pairing and support RNAP II transcription hubs (Fig. 3). Under these conditions, MVI functions as a chromatin crosslinker, increasing nuclear stiffness (Strom et al., 2021) by increasing chromatin rigidity and restricting mobility (Hochberg-Laufer and Shav-Tal, 2019). Chromatin dynamics during transcription have also been linked to nuclear integrity, whereby increased chromatin motion elevates the risk of nuclear blebbing and rupture (Berg et al., 2023). Future research must explore how myosins contribute directly to nuclear mechanics.

## Actin and myosin in cell mechanoadaptation

The role of nuclear actin and myosins in cell mechanoadaptation is an important contributor to cell plasticity. Rapid nuclear actin polymerisation initiated by integrins has been observed during cell spreading in mouse fibroblasts (Plessner et al., 2015). This highlights a clear interconnection between cellular reshaping and nuclear actin dynamics (McNeill et al., 2020; Mennens et al., 2017; Plessner et al., 2015; Sharili et al., 2016).

Moreover, in response to replication stress, nuclear F-actin levels increase both *in vitro* and *in vivo*. Nuclear F-actin boosts nuclear volume and sphericity, counteracting deformation (Lamm et al., 2020). Stimulation by F-actin and myosin II relocates stress-replication foci towards the nuclear membrane, mainly via movement along actin filaments, with additional contributions from diffusion. This shows that nuclear actin cooperates with myosin II to regulate chromatin organisation and nuclear architecture enabling adaption to stressful conditions.

Recently it has been shown that MVI stabilises stalled or reversed replication forks during replication stress (Shi et al., 2023). Using affinity probes to manipulate nuclear MVI levels, researchers showed that during DNA replication stress, MVI associates with stalled replication intermediates and interacts with Werner helicase interacting protein 1 (WRNIP1) to protect these structures from DNA2-mediated degradation. This stabilising effect prevents DNA damage-induced nuclear deformation (Kumar et al., 2024).

Studies on nuclear actin dynamics throughout mammalian cell division have revealed that F-actin is immediately assembled in the nucleus following mitosis (Baarlink et al., 2017). Specific inhibition of nuclear F-actin compromises nuclear expansion and hinders the formation of nuclear protrusions throughout the early G1 phase of the cell cycle. Chromatin density analysis in postmitotic nuclei with impaired nuclear F-actin assembly shows increased chromatin compaction.

As discussed above, NMI and MVI impact nuclear architecture through their involvement in gene transcription (Fili et al., 2017; Hari-Gupta et al., 2022; Philimonenko et al., 2004; Ye et al., 2008) and chromatin remodelling (Shahid-Fuente and Toseland, 2023; Shi et al., 2023). Nuclear myosins are particularly essential when cells experience high transcriptional load or rapid shifts in gene expression. This aligns with findings that MVI is overexpressed in various aggressive cancers, such as breast and prostate cancer (Dunn et al., 2006; Loikkanen et al., 2009; Puri et al., 2010; Zhan et al., 2023). MVI enhances transcription of genes involved in cell proliferation, migration, invasion and metastasis, promoting cellular plasticity and adaptation. Conversely, NMI, considered a global transcriptional regulator (Venit et al., 2020a), might function as a tumour suppressor. The NMI gene (*MYO1C*) is often mutated in various cancers (Oldfors et al., 2015; Visuttijai et al., 2016). NMI forms a complex with p53, activating the expression of the checkpoint regulator p21 (*CDKN1A*) to provide a transcriptional response to DNA damage, maintaining genome stability and nuclear architecture (Venit et al., 2020b).

The mechanisms through which external or internal cues influence nuclear actin and myosin to mediate cellular and nuclear plasticity remain largely unexplored. Understanding the role of nuclear actin and myosin in cell mechanoadaptation is essential to uncovering how cells modify their behaviour in response to changing conditions in both health and disease.

## Actin and myosin in pathology

Malformations of the nucleus, such as pathological nuclear stiffening, are often accompanied by nuclear actin disorganisation, a phenomenon observed in aging, cancer and stressed cells (Debaugnies et al., 2023; Gonzalez-Bermudez et al., 2022; Hoffman et al., 2020; Pendleton et al., 2003). Moreover, intranuclear actin rods are present in conditions, such as nemaline myopathy, anti-synthetase syndrome-induced dysimmune myopathy, and intranuclear rod myopathy (Domazetovska et al., 2007a,b; Labasse et al., 2022; Stenzel et al., 2015). These structures can also occur secondarily in certain disorders, including mitochondrial myopathies (Vandebrouck et al., 2010). The exact mechanism of nuclear actin rod formation is not fully understood, particularly with regards to their occurrence in pathologies with very different structural and metabolic defects. It remains unclear whether these rods are formed for a functional purpose or as by-products of regulatory defects. Nevertheless, their presence significantly reduces the mitotic index and adversely impacts cellular functions (Serebryannyy et al., 2016).

Nuclear actin plays a role in Hutchinson–Guilford progeria syndrome (HGPS), the most severe type of laminopathy. HGPS arises from aberrant splicing of the lamin A/C (*LMNA*) gene, resulting in the accumulation of a truncated form of lamin A (progerin), which is irreversibly farnesylated and toxic for cells. HGPS symptoms include nuclear envelope lobulation, thickened nuclear lamina, loss of heterochromatin from the nuclear periphery, clustering of nuclear pores, impaired nucleo-cytoskeletal coupling and nuclear stiffening (Goldman et al., 2004; Hale et al., 2008). These deformations increase the susceptibility of HGPS nuclei to mechanical stress (Dahl et al., 2006; Lammerding et al., 2004; Verstraeten et al., 2008). Notably, HGPS cells show decreased nuclear F-actin levels, and overexpression or boosted polymerisation of nuclear F-actin can restore their aberrant phenotypes (Takahashi et al., 2020). Lamin A also affects nuclear myosins, such as the isoform NMIβ, which is crucial for chromosome organisation during interphase (Mehta et al., 2010). Depletion of lamin A/C increases NMI localisation in the nucleus (Ranade et al., 2019). Similarly, farnesylated progerin impairs NMIβ distribution, whereas farnesyltransferase inhibition restores NMIβ localisation and repositions chromosome territories within interphase nuclei (Mehta et al., 2011). These findings suggest a link between the nuclear lamina, nuclear actin and myosin in regulating chromatin architecture and mechanosensing in pathology. However, the contributions of nuclear pools of actin and myosin to HGPS aetiology remain poorly understood and require further studies.

Impaired nuclear stiffness and altered lamin expression are observed in many cancer types, making them potential prognostic markers (Hudson et al., 2007; Sun et al., 2010; Tilli et al., 2003; Wu et al., 2009). Although mutations in the actin β (*ACTB*) gene are infrequent in cancer generally, their prevalence increases in highly invasive cancers, such as metastatic melanoma and diffuse large B-cell lymphoma (https://www.cbioportal.org/). Aberrated nuclear and cellular actin levels can increase the metastatic potential of cancer cells by making nuclei more elastic and compressible, facilitating penetration of surrounding tissues and blood vessels (Lei et al., 2021; Zwerger et al., 2011). Conversely, compressive solid stresses in metastatic breast cancer lymph nodes increase resistance to lymphocyte infiltration and accelerate metastatic cell growth (Jones et al., 2021). These observations show that changes in nuclear biophysical properties vary across cancer types and depend on tumour heterogeneity and stage (Zwerger et al., 2011). Although nuclear actin mislocalisation appears to influence cancer cell fate, its exact role in carcinogenesis remains intertwined with more complex spatiotemporal regulatory mechanisms. It is important to determine whether nuclear actin accumulation drives pathology onset and progression or merely reflects underlying disturbances in cellular physiology.

Mutations in nuclear myosins, such as MVI, are associated with cardiomyopathy (Hegan et al., 2015; Karatsai et al., 2023; Mohiddin et al., 2004), impaired skeletal muscle function (Lehka et al., 2020, 2022), spermiogenesis defects (Zakrzewski et al., 2020) and cancer (Dunn et al., 2006; Yoshida et al., 2004). However, the involvement of nuclear myosins in the pathomechanisms of these disorders remains understudied. In certain cancers, such as breast, prostate and ovarian cancers, MVI is overexpressed, with isoform enrichment biased towards variants readily recruited to the nucleus (Fili et al., 2017; Puri et al., 2010; Yoshida et al., 2004; Zhan et al., 2023). Whether the nuclear pool of MVI is specifically enhanced in these tumours remains to be directly studied.

## Conclusions and perspectives

Recent developments in chromatin organisation research support that the nuclear interior has an important role in the mechanical properties of this organelle. In response to internal and external stimuli, chromatin can acquire different physical states, ranging from liquid and gel-like to rigid. These physical states, combined with the epigenetic regulation of chromatin compaction, contribute to the overall stiffness and morphology of the nucleus (Maeshima et al., 2016; Salari et al., 2022; Strickfaden et al., 2020).

Mounting evidence supporting the role of nuclear actin and myosins in chromatin organisation raises new questions about their involvement in regulating nuclear mechanics (Cook et al., 2020; Dos Santos and Toseland, 2021; Percipalle and Vartiainen, 2019; Sen et al., 2024; Ulferts et al., 2024). These proteins function as hubs, anchors and cross-linkers, directly reshaping the nucleus by scaffolding and compartmentalising chromatin. Both nuclear actin and myosin promote the formation of RNAP II clusters with transcription factors (Cisse et al., 2013; Hari-Gupta et al., 2022; Wei et al., 2020). Such RNAP II hubs decrease chromatin mobility, potentially impacting local nuclear stiffness (Ashwin et al., 2020; Nagashima et al., 2019). The formation of these structures has been linked to phase separation (Boehning et al., 2018; Flores-Solis et al., 2023). Investigating the involvement of nuclear actin and myosin in phase separation represents an interesting avenue for future research. Nuclear myosins also play an important role in the DNA damage response, which affects nuclear mechanics (Cook and Toseland, 2021). DNA damage response activation causes nuclear softening and chromatin decondensation, accompanied by increased molecular diffusion within the nucleus (dos Santos et al., 2021).

Additional findings suggest that nuclear myosins and actin might serve not only as structural scaffolds but also as participants in long-distance chromatin reorganisation (Dundr et al., 2007; Zorca et al., 2015). For instance, MVI movement on chromatin has been proposed to resemble its cytoplasmic motility and depends on the ATPase activity of the protein (Große-Berkenbusch et al., 2020 preprint). These functions might have implications for the long-range movement of chromatin, which can impact nuclear mechanics. Furthermore, accumulating evidence indicates that nuclear motors can directly and indirectly affect nuclear mechanics and that nuclear myosins and actin are functionally linked to the nuclear lamina (Holaska and Wilson, 2007; Mehta et al., 2008; Mishra and Levy, 2022).

Overall, well-characterised proteins with known impacts on cell mechanobiology are present in the nucleus and elicit multiple functions. Therefore, future studies should investigate the potential roles of these proteins in nuclear force transduction. A particularly intriguing remaining question concerns how nuclear myosins and actin interact to influence nuclear morphology. Answering this question requires analysing the mechanical contributions of the nuclear interior, isolated from the perinuclear cytoskeleton, which is a challenging task. However, advances in experimental techniques and the integration of different biological methods are making this goal increasingly achievable (Nelsen et al., 2020). A comprehensive understanding of the function of molecular motors in the nucleus is urgently needed. Future research should focus on their relationship in regulating nuclear mechanics, providing a holistic view of their functional roles.
